# Habitat differentiation within the large-carnivore community of Norway's multiple-use landscapes

**DOI:** 10.1111/j.1365-2664.2008.01527.x

**Published:** 2008-10

**Authors:** Roel May, Jiska van Dijk, Petter Wabakken, Jon E Swenson, John DC Linnell, Barbara Zimmermann, John Odden, Hans C Pedersen, Reidar Andersen, Arild Landa

**Affiliations:** Norwegian Institute for Nature ResearchTungasletta 2, NO-7485 Trondheim, Norway; Faculty of Forestry and Wildlife Management, Hedmark University CollegeEvenstad, NO-2480 Koppang, Norway; Department of Ecology and Natural Resource Management, Norwegian University of Life SciencesPO Box 5003, NO-1432 Ås, Norway; Museum of Natural History and Archaeology, Norwegian University of Science and TechnologyNO-7491 Trondheim, Norway

**Keywords:** brown bear, elevational gradients, Eurasian lynx, grey wolf, habitat and predation patterns, intra-guild competition, regional zoning of large carnivores, species co-existence, wolverine

## Abstract

1. The re-establishment of large carnivores in Norway has led to increased conflicts and the adoption of regional zoning for these predators. When planning the future distribution of large carnivores, it is important to consider details of their potential habitat tolerances and strength of inter-specific differentiation. We studied differentiation in habitat and kill sites within the large-carnivore community of south-eastern Norway.
2. We compared habitat selection of the brown bear *Ursus arctos* L., Eurasian lynx *Lynx lynx* L., wolf *Canis lupus* L. and wolverine *Gulo gulo* L., based on radio-tracking data. Differences in kill site locations were explored using locations of documented predator-killed sheep *Ovis aries* L. We modelled each species’ selection for, and differentiation in, habitat and kill sites on a landscape scale using resource selection functions and multinomial logistic regression. Based on projected probability of occurrence maps, we estimated continuous patches of habitat within the study area.
3. Although bears, lynx, wolves and wolverines had overlapping distributions, we found a clear differentiation for all four species in both habitat and kill sites. The presence of bears, wolves and lynx was generally associated with rugged, forested areas at lower elevations, whereas wolverines selected rugged terrain at higher elevations. Some degree of sympatry was possible in over 40% of the study area, although only 1·5% could hold all four large carnivores together.
4. *Synthesis and applications*. A geographically differentiated management policy has been adopted in Norway, aimed at conserving viable populations of large carnivores while minimizing the potential for conflicts. Sympatry of all four carnivores will be most successful if regional zones are established of adequate size spanning an elevational gradient. High prey densities, low carnivore densities, low dietary overlap and scavenging opportunities have most probably led to reduced competitive exclusion. Although regional sympatry enhances the conservation of an intact guild of large carnivores, it may well increase conflict levels and resistance to carnivore conservation locally.

## Introduction

During the last century, habitat fragmentation and increased human pressure have reduced populations of large carnivores throughout the world (Woodroffe 2000; Sunquist & Sunquist 2001). Although large carnivores are able to persist in multiple-use landscapes, many mammalian carnivores possess characteristics that may make them particularly vulnerable to landscape changes (Woodroffe & Ginsberg 1998). Carnivore species may react differently to fragmentation however, due to differences in behaviour and ecology (Sunquist & Sunquist 2001; Crooks 2002).

Apart from direct competition for prey, possible sympatry of multiple carnivore species also depends on intra-guild competition and interference. Intra-guild competition is fiercer with higher dietary or spatial overlap, is often asymmetrical and may have strong effects on the population dynamics of the subordinate competitor (Creel, Spong & Creel 2001; Heithaus 2001). Linnell & Strand (2000) hypothesized that interference may reduce population growth through temporal and spatial avoidance, changes in foraging efficiency, or direct killing, irrespective of dietary and habitat overlap. Whereas intra-guild competition is thought to be density-dependent, the degree of intra-guild interference is thought to depend on body-size differences (Buskirk 1999). Intra-guild competition and interference may ultimately lead to habitat differentiation (i.e. competitive exclusion). In addition, subordinate predators may also be suppressed in the absence of scavenging opportunities from top predators (Buskirk 1999).

Four species of large carnivores are present in Scandinavia: the brown bear *Ursus arctos*L., grey wolf *Canis lupus* L., Eurasian lynx *Lynx lynx*L. and wolverine *Gulo gulo*L. The conservation of large carnivores in Scandinavia is dependent upon co-existence with humans in a multiple-use landscape. The natural recovery of carnivore populations, however, has led to increased conflict. The main causes of conflict are their depredation on semi-domestic reindeer *Rangifer tarandus* L. throughout the year in Fennoscandia, and on free-ranging domestic sheep *Ovis aries* L. during summer, primarily in Norway (Swenson & Andrén 2005). Although most predation on reindeer is caused by wolverines and lynx, all large carnivores in Norway kill free-ranging sheep. This has led to the adoption of a geographically differentiated management policy (i.e. zoning of large carnivores) aimed at conserving viable populations of large carnivores while minimizing the potential for conflicts (Ministry of Environment 2003; Linnell *et al*. 2005). When planning the future distribution of large carnivores, it is important to consider details of their potential habitat tolerances, and strength of differentiation among the four species. The present population goals for large carnivores in Norway are specified for eight management regions (Committee on Energy and Environment 2004). The large-carnivore region of Hedmark County is the only region that has populations of all four large-carnivore species. In this region, we analysed large-carnivore habitat use based on radio-telemetry and location of sheep kill sites. Compared to kill sites, radio-telemetry locations represent a wide spectrum of habitat used by a carnivore. Radio-telemetry locations include resting places, kill sites of wild and domestic ungulates including sheep, as well as movements between kill sites and resting places. However, kill sites represent the main cause of the conflict between large carnivores and human interests. Our initial expectation was that bears, wolves and lynx would have broadly similar patterns of occurrence (forest-dwelling species). However, through the effects of intra-guild competition and interference, they were expected to show differentiation in habitat use. By contrast, we expected the wolverine to be clearly differentiated in habitat, due to the combined effects of their perceived susceptibility to fragmentation and avoidance of other carnivores. Likewise, we expected that potential avoidance of other carnivore species would affect the distribution of sheep kill sites; especially in the lynx and wolverine as subordinate predators.

## Materials and methods

### study area

**Fig. 1:**
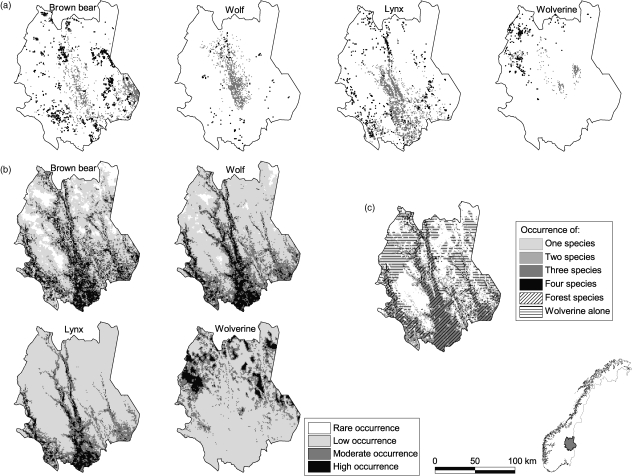
(a) Presence maps for four large-carnivore species within the study area in south-eastern Norway (see corner). Presence pixels from radio-tracking data are given in grey; locations of sheep killed by each carnivore species are given as black dots. (b) Occurrence maps for each species; probability distributions were based on species-specific resource selection function models (Fig. 2). (c) Possible sympatry based on the overlap of moderate- and high-occurrence classes for each species.

Norway is the country in mainland Europe with the lowest human population density (*c*. 12 km^−2^) and with large continuous areas of semi-natural landscapes. Despite the low human density, wilderness areas have declined dramatically in the last century through resource extraction (i.e. livestock grazing, hunting, timber logging), infrastructure development (i.e. roads, recreational cabins and hydropower plants), and recreation. Our study area (18 374 km^2^) was located in south-eastern Norway. It consists of 10 municipalities in the northern parts of Hedmark County and three bordering municipalities in Oppland County (see corner Fig. 1), and was centred on lake Storsjøen (latitude 61°27′, longitude 11°18′). The river Glomma and the adjacent national highway RV3 run from north to south in the centre of the study area. The landscape consists of boreal forests interspersed with low mountain ranges. Areas above tree line, at 900–1000 m above sea level, are mainly found in the west and north of the study area. Infrastructure is mainly found in the south and west of the study area, and in the valley bottoms. All four large-carnivore species exist within the study area and were estimated by the national large-carnivore monitoring programme at 14–17 wolves (three to four packs or scent-marking pairs), 20–30 wolverines and 31–37 lynx (Brøseth & Andersen 2004; Brøseth, Odden & Linnell 2004; Wabakken *et al*. 2004). The total number of bears was estimated at nine to 13 for southeast Norway (Østlandet) (Swenson *et al*. 2003). The populations of all four species are in a natural re-colonizing stage, with the bear population in particular being dominated by males. Bears and lynx were already present before the start of this study (1988) and have expanded their range from the (north)east and from the (south)east, respectively. Wolves re-colonized the study area in 1998 from the (south)east; wolverines followed the year after from the northeast and west. At present, all four species occur throughout the study area. The average winter densities of potential large prey species are 0·9 km^−2^ and 0·8 km^−2^ for moose *Alces alces* L. and roe deer *Capreolus capreolus* L., respectively (Solberg *et al*. 2003). However, roe deer are distributed less evenly over the area than moose. Other potential ungulate prey species are red deer *Cervus elaphus*L. and wild reindeer. Moreover, semi-domestic reindeer are herded in the north-eastern two municipalities of the study area. Potential small prey are tetraonids and other bird species, medium-sized and small rodents and insectivores, as well as medium-sized and small carnivores. Throughout the study area, with disjoint distribution and at highly variable densities, free-ranging, and mostly unattended domestic sheep and cattle *Bos taurus* L. graze in the forests and low mountain ranges during the summer (June–September) (Zimmermann, Wabakken & Dötterer 2003).

**Fig. 2:**
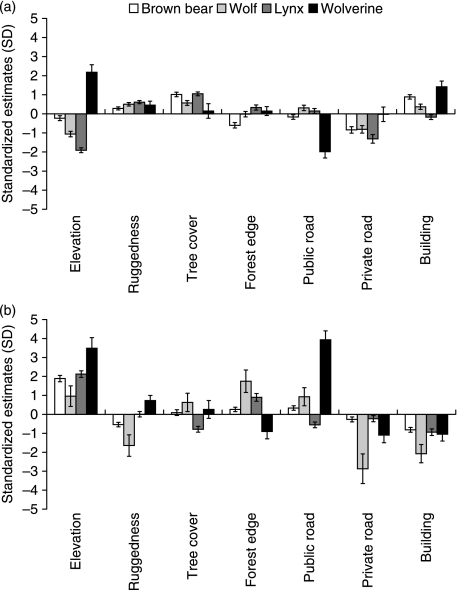
Standardized estimates (± SD) of the resource selection functions for (a) habitat selection relative to available habitat within the study area, and (b) location of kill sites relative to selected habitat. The last four covariates are distance measures.

### study design and spatial scale

The scale (i.e. grain/resolution and domain/extent) of investigation of the differentiation in habitat tolerances among guild members is important, as ecological processes can occur at different spatio-temporal scales, which influence the strength of habitat preferences (Boyce 2006). Our spatially, but not temporally, overlapping data sets (Table 1) on the large-carnivore guild in one specific region of Norway best fit a landscape approach. To address differentiation among wide-ranging large-carnivore species, the resolution need not be very fine; a coarser grain will reduce intra-specific spatial heterogeneity at finer resolutions leaving the inter-specific differences under study. However, the extent should be large enough to encompass the regional dynamics of the large-carnivore community in multiple-use landscapes. We therefore chose to study patterns of use on the landscape using a grain of 1 × 1 km resource units (pixels), and investigated habitat differentiation within the large-carnivore guild by comparing selection of geographical ranges among the species within the study area (first order selection, Johnson 1980).

**Table 1:** Sampling statistics of the radio-tracked large carnivores and predator-killed sheep in south-eastern Norway

	Brown bear	Wolf	Lynx	Wolverine
Statistics habitat				
Collection period	1988–2004	2001–2005	1995–2002	2003–2004
Collection methods (collar type)	VHF, GPS	GPS	VHF, GPS	GPS
No. of individuals	20	4*	16	4
Adult females	5	2	10	3
Adult males	15	2	6	1
Individuals per year (± SD)	4·9 ± 1·4	2·6 ± 0·9	7·6 ± 4·6	3·5 ± 0·7
Total radio fixes (> 24 h apart)	2194	2780	3681	453
No. of radio fixes per individual (± SD)	110 ± 139	498 ± 305	230 ± 144	227 ± 88
No. of habitat pixels (Fig. 1)	1169	874	1761	265
Statistics kill sites				
No. of sheep carcasses	1558	416	861	364
No. of kill site pixels (Fig. 1)	760	102	462	218

* Two alpha pairs of two packs.

### topographic and habitat covariates

Habitat differentiation among the four large-carnivore species was investigated using seven habitat covariates: elevation, terrain ruggedness, percentage tree cover, distance to the forest edge, and distance to the nearest public road, private road and building. Elevation was obtained from a 100 × 100 m Digital Elevation Model (DEM; Norwegian Mapping Authority). Terrain ruggedness was calculated by taking the square root of the sum of squared differences in elevation of each pixel in the 100 × 100 m DEM to its eight neighbours, thus rendering a terrain ruggedness index (Riley, DeGloria & Elliot 1999). Percentage tree cover was obtained from a MODIS map (Hansen *et al*. 2002). The four distance measures were obtained from digital 1:50 000 topographic maps (Norwegian Mapping Authority). Distances to the forest edge were negative inside and positive outside the forest. All maps were finally converted into overlapping 1 × 1 km pixel grids.

### data sets

The study was based on radio-tracking adult individuals within research projects on large carnivores (Table 1). Only locations more than 24 h apart were used in order to reduce autocorrelation (Otis & White 1999) and standardize between GPS and VHF data (i.e. several positions per day vs. up to one position per day, respectively). As the data were collected during different time periods, this study renders insight into spatial but not necessarily temporal sympatry of the four large carnivores.

Location of kill sites was assessed using locations of documented predator-killed sheep falling within the boundaries of the study area from the period 1994–2004 (Fig. 1a). To receive compensation for losses suffered by predators, it is economically important to the owners of free-ranging sheep to intensively search for carcasses throughout the summer grazing season (~100 days yr^−1^). Carcasses are examined by trained personnel of the State Nature Inspectorate, who record the location and determine the species of the predator, based on well-documented species-specific kill patterns through necropsy (Landa 1999). Although the locations of sheep kills found are likely to be biased towards ease of human detection, this can be expected to be irrespective of carnivore species.

### modelling and statistical analyses

All statistics were performed in r 2·5·1 (r Development Core Team 2007), the geographical analyses were performed in ArcView 3·3 and Spatial Analyst extension (ESRI Inc., Redlands, CA, USA). For each species, we transformed the set of radio-tracking locations and killed sheep into presence maps, where each 1 × 1 km pixel indicated whether or not it included one or more locations (Fig. 1). This large scale minimizes unwanted spatial autocorrelation and pseudo-replication effects. We expected a pseudo-replication effect for the members of the two wolf packs while travelling together, and for animals that were tracked over several years. Also, large carnivores, especially bears and wolves, often kill several sheep during one attack. Here we assumed that the individuals used in this study represented the resource selection of the species. Intra-specific variation was found to be insignificant compared to inter-specific variation (see Supplementary Material Appendix S1).

We modelled each species’ habitat selection relative to availability on a landscape scale, and each species’ location of kill sites relative to its habitat used (i.e. presence pixels), using logistic regression to estimate coefficients in exponential resource selection functions (Manly *et al*. 2002):

eqn 1 $$w(x)=\exp(\beta_{0}{+\beta }_{\text{1}}ċX_{1}+\beta_{2}ċX_{2}+...+\beta_{n}ċX_{n})$$

with β*_i_* as the model coefficient of the *i*^th^ of *n* habitat covariates, *X_i_*. Availability for habitat selection was considered to be the same for all species, and was based on a ‘presence’ map generated from a data set of 2500 points randomly spread throughout the study area following the same procedure as mentioned above (rendering 2311 availability pixels). Because the focus of this study was to elucidate habitat differentiation among large carnivores, we present the full models only. To evaluate predictive success of the resource selection function models we used the *k*-fold cross-validation procedures as proposed by Boyce *et al*. (2002). Cross-validated Spearman-rank correlations were calculated between 10 resource selection function bin ranks and area-adjusted frequencies for five model ‘test-training’ sets.

The resource selection functions for habitat selection were for each cross-validation set projected spatially on each 1 × 1 km cell across the study area to generate maps of the relative probability of occurrence for each species. For ease of interpretation, we classified the relative probabilities of occurrence into four broad classes: rare- (< −1σ), low- (−1σ − µ), moderate- (µ − 1σ) and high- (> 1σ) occurrence habitats. To gain better insight into the scale of our study area vs. necessary scales for regional zoning, we calculated the degree of overlap among species for each cross-validation set based on the distribution of moderate- and high-occurrence habitats for each species (i.e. pixels with relative probability > µ). Finally, we calculated the distribution of patch sizes for each species and degree of overlap, based on occurrence maps produced from the averages over the set of five cross-validation maps. Continuous patches were identified using the Patch Analyst 2·2 extension (Rempel 2000), after smoothening the occurrence maps using a 3 × 3 moving window majority filter.

We estimated the overall strength of differentiation among species both in habitat use and location of kill sites by calculating the multivariate distance over the standardized resource selection functions coefficients. Standardized coefficients allow comparisons of the relative influence of resources on selection, regardless of the measurement scale quantifying the resource (Marzluff *et al*. 2004). The standardized coefficients for each resource covariate 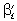 were estimated as:

eqn 2
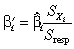


where β_*i*_ is the maximum-likelihood estimate of the coefficient for resource *i*; 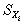 is the standard deviation of the values of resource *i*; and *S*_resp_ is the estimate of the standard deviation of the response values. The standardized standard errors of the coefficients 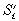 were calculated in a similar fashion. The multivariate distance between two species *j* and *k* was calculated as:

eqn 3
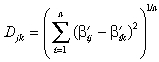


We incorporated the uncertainty from the resource selection functions by calculating the average multivariate distances from 1000 iterated random draws from a distribution with mean 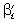 and standard error 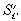 We used a linear stretch to scale the multivariate distances between –1 and +1 for totally differentiated and identical selection, respectively:

eqn 4
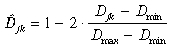


Finally, we performed multinomial logistic regression (Hosmer & Lemeshow 2000) on the combined presence data over all species (separately for habitat and kill sites) to investigate each species’ degree of differentiation in habitat use (or location of kill sites) relative to habitat (or kill sites) used by the other species, and determine which covariates they differed, and how strongly. The species were taken as a categorical dependent variable (1 = bear, 2 = wolf, 3 = lynx, 4 = wolverine). By taking each species as a reference category in an iterative way, each unique species combination could be compared.

## Results

### habitat use and location of kill sites

All four habitat models had good predictive performance, given the significant Spearman-rank correlations across the five cross-validation sets (bear: 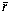 = 0·980 ± 0·010 (SD), *P* < 0·001; wolf: 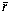 = 0·950 ± 0·026, *P* < 0·001; lynx: 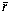 = 0·979 ± 0·014; *P* < 0·001; wolverine: 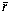 = 0·859 ± 0·097, *P* < 0·001). The models explained 13–14% of the deviance for bears, wolves and wolverines, and 40% for lynx (Nagelkerke's *R*^2^ of 0·139, 0·129, 0·142 and 0·402, respectively). The resource selection functions for bears, wolves and lynx indicated that the presence of these species was generally associated with rugged, forested areas at lower elevations, and relatively close to private roads (Fig. 2a, Supplementary Material Table S1). Of these species, lynx preferred the lowest elevations, the densest forests, and kept closest to infrastructure. Wolverines, on the other hand, selected rugged terrain at higher elevations and away from buildings but closer to public roads. They did not show any selection for tree cover or private roads.

The kill site models for bears, wolves and lynx had good predictive performance and explained 16–22% of the deviance (bear: 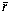 = 0·919 ± 0·048 (SD), *P* < 0·001, Nagelkerke's *R*^2^ = 0·201; wolf: 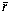 = 0·804 ± 0·081, *P* < 0·001, *R*^2^ = 0·163; lynx: 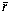 = 0·932 ± 0·018; *P* < 0·001, *R*^2^ = 0·215). The kill site model for wolverines had a lower, but significant, Spearman-rank correlation and explained over 50% of the deviance (wolverine: 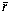 = 0·601 ± 0·159 (SD), *P* < 0·05, Nagelkerke's *R*^2^ = 0·570). Sheep kill sites were for all four species found at higher elevations and closer to private roads and buildings compared to their selected habitat (Fig. 2b, Supplementary Material Table S1). The three forest-dwelling species killed sheep in less rugged terrain and farther from forest edges; opposite effects were found for the wolverine. All species, except lynx, killed sheep farther from public roads.

### patterns of intra-guild distribution

**Fig. 3:**
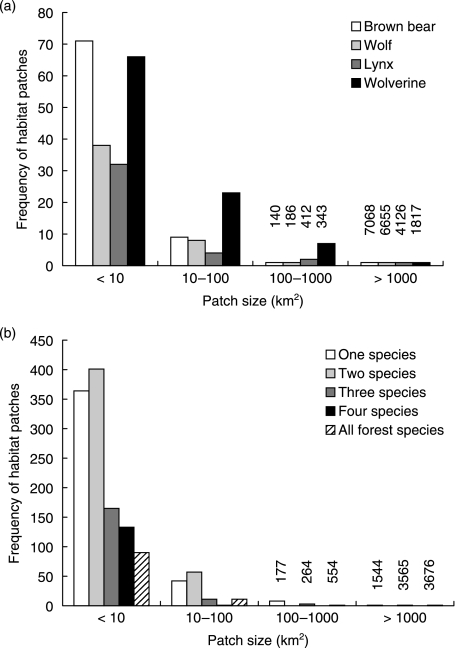
Size distribution of habitat patches for (a) four large-carnivore species identified using resource selection functions (Fig. 1b) and (b) degree of overlap in south-eastern Norway (Fig. 1c). For the highest two categories the average patch size is given.

A clear distinction can be seen between the distributions of wolverines vs. the three forest-dwelling carnivore species (Fig. 1b). Whereas wolverine presence was most probable in the more mountainous northwest of the study area, the presence of the other three species was more distributed in the south and along the Glomma Valley running from north to south in the centre of the study area. Using a minimum threshold of moderate occurrence, 7490 km^2^ ± 87 (SD) of the study area was defined as suitable for bears, and 7126 ± 124, 5214 ± 64 and 5418 km^2^ ± 117 were classified as suitable for wolves, lynx and wolverines, respectively. The mean patch size for bear, wolf, lynx and wolverines were 93 ± 780 (SD), 149 ± 959, 133 ± 664 and 54 ± 210 km^2^, respectively. The wolverine both had a high amount of small patches (< 10 km^2^) and the smallest average patch size >1000 km^2^ (Fig. 3a).

We calculated the degree of overlap among species and patch sizes based on the distribution of moderate- and high-occurrence habitats in the occurrence maps for each species (Fig. 1c). Using a minimum threshold of moderate occurrence, 4893 km^2^ ± 141 (SD) of the study area could hold only one species, whereas 2612 ± 50, 4671 ± 72 and 280 ± 38 km^2^ were classified as suitable for two, three and four species, respectively. In total, 4496 km^2^ ± 47 (SD) of the study area could hold all three forest species. Again, the clear distinction between sympatry of the three forest-dwelling species and the wolverines is clear (Fig. 1c), with a 60–99% overlap between bears, wolves and lynx and a 5–29% overlap with wolverines (Table 2). The mean patch sizes for overlap of one, two, three, four species and overlap of the three forest species were 12 ± 80 (SD), 5 ± 8, 28 ± 269, 2 ± 1 and 47 ± 365 km^2^, respectively. Both the overlap of three species and of the forest-dwelling species had a relatively low amount of small patches (< 10 km^2^) and large average patch sizes > 1000 km^2^ (Fig. 3b).

**Table 2:** Proportional degree of overlap (± SD) in distribution between species based on the distribution of occurrence for each species (pixels with a probability higher than the mean), averaged over five cross-validation sets

	Proportional overlap with distribution of			
	
Species distribution	Brown bear	Wolf	Lynx	Wolverine
Brown bear		0·802 ± 0·018	0·601 ± 0·008	0·209 ± 0·014
Wolf	0·843 ± 0·017		0·726 ± 0·009	0·109 ± 0·009
Lynx	0·863 ± 0·012	0·992 ± 0·010		0·055 ± 0·009
Wolverine	0·289 ± 0·018	0·143 ± 0·011	0·053 ± 0·008	

### differentiation in habitat and kill sites

Overall, wolverines differed in their habitat use compared to the three forest-dwelling carnivore species (Table 3). Bears–wolves and wolves–lynx selected similar habitat, but no differentiation was found between bears and lynx. The overall differentiation in location of kill sites showed a clear difference for wolverines compared to the three forest-dwelling species. Although no differentiation was found for bears–wolves and wolves–lynx, bears and lynx killed sheep in similar habitat (Table 3).

**Table 3:** Strength of differentiation in habitat use and location of kill sites between species as measured by the multivariate distances between the standardized partial regression coefficients, given in Fig. 2. Negative mean values indicate differentiation and positive values similar use/location. When the 95% CI includes zero; neither could be determined. Significant results are given in bold

Species pairs		Mean	SD	95% CI
Habitat use				
brown bear	wolf	**0·561**	0·112	0·341–0·781
brown bear	lynx	0·114	0·076	−0·034–0·263
brown bear	wolverine	**−0·281**	0·117	−0·511––0·051
wolf	lynx	**0·577**	0·116	0·349–0·804
wolf	wolverine	**−0·513**	0·104	−0·717––0·309
lynx	wolverine	**−0·735**	0·090	−0·911––0·559
Kill sites				
brown bear	wolf	−0·181	0·176	−0·526–0·163
brown bear	lynx	**0·595**	0·098	0·403–0·786
brown bear	wolverine	**−0·329**	0·112	−0·549––0·110
wolf	lynx	−0·303	0·156	−0·608–0·002
wolf	wolverine	**−0·627**	0·132	−0·886––0·368
lynx	wolverine	**−0·512**	0·102	−0·712––0·313

**Fig. 4:**
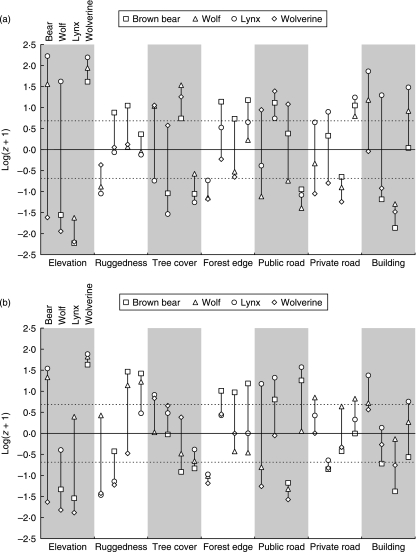
Multinomial logistic regression results for comparisons of habitat use (a) and location of kill sites (b) among four carnivore species in south-eastern Norway. The z-scores given represent the strength of differentiation between species. Each species’ degree of differentiation is shown relative to the other three species for each covariate. The sign indicates the direction of the effect. Values larger than ± 0·685 (dotted lines) indicate significant differentiation.

Multinomial logistic regression indicated a clear differentiation in use of habitat covariates among all four species (Nagelkerke's *R*^2^ = 0·318, Fig. 4a). The strongest differentiation in preference was found for elevation. Lynx were found at the lowest elevations, followed in rising elevation by wolves, bears and wolverines (Fig. 4a, Supplementary Material Table S1). The bear was found in less rugged terrain and closer to forest edges than the other three species. Also, a clear effect in differentiation was found for tree cover and infrastructure. The lynx preferred pixels with a higher percentage of tree cover, and closer to private roads and buildings than the bear and wolf. The wolverine was found in more open areas far from private roads and buildings. The wolf and wolverine also differentiated from bear and lynx in their proximity to public roads.

Multinomial logistic regression on the locations of predator-killed sheep indicated a clear differentiation in kill sites among species (Nagelkerke's *R*^2^ = 0·485, Fig. 4b, Supplementary Material Table S1). As for the differentiation in habitat, elevation of kill sites had a similar strong differentiating effect; except in the case of wolf–lynx kill sites. For these two species, ruggedness and distance to public roads at kill sites differed most. Lynx and wolverines killed sheep in more rugged terrain than bears and wolves. Wolverines killed sheep in more open areas, whereas bears chose more forested sites closer to forest edges. Lynx stayed closer to public roads and buildings than the other species. Wolves killed sheep closer to private roads than bears and wolverines.

## Discussion

The results from this study indicate that the three forest-dwelling large-carnivore species, the lynx, wolf and bear, had relatively similar habitat preferences. All three species selected rugged, forested areas at lower elevations. In contrast, the wolverine selected open, rugged terrain at higher elevations and killed sheep in similar terrain, but farther from infrastructure. This result fits well with the perception that the wolverine is a carnivore of remote alpine regions (May *et al*. 2006; Copeland *et al*. 2007). Wolverines overlapped most with bears and least with lynx (cf. Carroll, Noss & Paquet 2001). Within the study area, sympatry of wolverines with the three forest-dwelling carnivores appears to depend on the availability of mountain ranges as a spatial refuge. Wolverines in our study area, however, depend highly on moose carcasses in their diet both from hunting leftovers and wolf-kills (van Dijk, Gustavsen, Mysterud, May, Flagstad, Brøseth, Andersen, Andersen, Steen & Landa 2008). The wolf is likely to be least affected by intra-guild aggression; rather it may instigate it (i.e. intra-guild predator, Palomares & Caro 1999). Additionally, wolves may facilitate wolverines with scavenging opportunities (Landa *et al*. 1997; Wilmers *et al*. 2003), which may enhance sympatry (Landa & Skogland 1995).

Despite their similar potential distribution patterns, the three forest-dwelling species had clear differences in habitat and kill sites. Bears preferred less rugged and higher elevation terrain than wolves and lynx, and chose more forested kill sites closer to forest edges. Although both wolves and bears feed on moose (Sand *et al*. 2005; Swenson *et al*. 2007), aggressive exploitative competition is not likely to be of significance because of the omnivorous diet of bears (Dahle *et al*. 1998) and low densities of both bears and wolves in the study area. Bears may also benefit to some extent from the presence of other predators through increased scavenging opportunities (MacNulty, Varley & Smith 2001; Wilmers *et al*. 2003). Our study showed that wolves and lynx differed least in habitat use. However, lynx used denser forests at lower elevations and killed sheep in more rugged terrain than wolves, which may reflect differences in hunting techniques (i.e. stalking vs. chase hunt), different habitat preference during hunting and avoidance of intra-guild predation. Also, lynx prey mainly on roe deer and small game (Odden, Linnell & Andersen 2006) in our study area.

In this study, we modelled carnivore selection for habitat and location of kill sites. Although resource selection in carnivores will also depend on local differences in wild ungulate densities and probability of encounters (Hebblewhite, Merrill & McDonald 2005), no such fine-scale prey data were available. As expected, kill sites were biased towards higher-lying, more open areas closer to private roads and buildings, indicative of sheep grazing preferences and ease of human detection. Kill sites may however be biased by specific sex or age groups of large carnivores (e.g. lynx males: Odden *et al*. 2006; wolverine females: Landa *et al*. 1997; young dispersers of all species). Also, most bear kill sites were found in the lower occurrence classes, which is likely to be due to the bears’ non-territorial behaviour. Still, understanding differentiation in kill sites among species provides important information for future management of depredation conflicts. Overall, elevation had the strongest differentiating effect both on selection of habitat and location of kill sites in all four large carnivores. The presence of guild members may well have resulted in elevational shifts in their respective distributions to avoid aggressive interactions. It is likely, however, that high prey densities, low large-carnivore densities (due to management actions) and low dietary overlap have led to a situation with reduced competitive exclusion (cf. Heithaus 2001).

In a broader regional context, our study area encompasses similar habitat/land use compositions and prey densities as that found in large stretches of southern Norway and central Sweden, and has a carnivore management regime comparable to other regions in Norway. The spatial extent of regional planning depends on the scale at which population processes are occurring. Our estimates of available patches for large carnivores inside the entire study area may render insight into the minimum area required for viable populations and scale of regional zoning (cf. Mech 1995). Large carnivores are known to be vulnerable to anthropogenic disturbance (e.g. May *et al*. 2006; Nelleman *et al*. 2007). Our modelling indicates that wolverines were most sensitive to fragmentation of habitat, given the high amount of small disjointed patches. For the three forest-dwelling species, a continuous geographical unit could be delimited in the south of the study area (see Fig. 1c).

To explain present distributions, habitat preferences and differentiation among Scandinavian large carnivores, historical management and the role of humans as a top predator in multiple-use ecosystems should not be underestimated. The main reason for the decline in large-carnivore populations in Scandinavia was human-induced mortality caused by (over)exploitation, persecution because of livestock/game conflicts, and fear (Swenson *et al*. 1995; Linnell *et al*. 2002; Linnell *et al*. 2005). The current forest-dominated distribution of bears in Scandinavia is based on re-colonization from remnant populations that survived in remote areas in Sweden (Swenson *et al*. 1995). Similarly, centuries of heavy persecution of wolverines all over Norway until 30 years ago may partly explain the habitat preferences and more remote distribution of wolverines found at present (Landa & Skogland 1995; May *et al*. 2006). Although the wolf was functionally extinct in the late 1960s after decades of intensive persecution, they have now re-established in south-central Scandinavia (Wabakken *et al*. 2001). After having been reduced to very low levels in the mid-20th century due to unregulated hunting and high bounties, changes in management have led to a recovery of lynx population in Scandinavia (Andrén *et al*. 2002).

Although sympatry of two or more species was possible in over 40% of the study area, only 1·5% was suitable for all four species together. Sympatry of all three forest carnivores was possible in one-quarter of the study area. Successful regional zoning of all four carnivores may therefore rely on establishing zones of adequate size spanning an elevational gradient. Zoning of all four species may enhance the conservation of an intact guild of large carnivores in the boreal forest ecosystem (Wabakken 2001). On the other hand, fostering sympatry of all four species may well increase conflict levels and resistance to carnivore conservation locally (Wabakken 2001; Linnell *et al*. 2005). These conflicts may be reduced by discouraging extensive sheep husbandry (Zimmermann *et al*. 2003; Milner *et al*. 2005), employing effective preventive and mitigation measures required for adequate compensation schemes, promoting different lifestyles and livelihood (e.g. ecotourism and outdoor recreation), and also allowing for limited control (Linnell *et al*. 2005; Swenson & Andrén 2005). However, the social context (non-material nature) of many of the large-carnivore conflicts in Norway should never be forgotten (Skogen 2003). Our study results may provide guidance to managers attempting to design regional-scale zoning to facilitate recovery of large carnivores in Scandinavia.

## Supplementary material

The following supplementary material is available for this article:

**Appendix S1.** Intra- vs. inter-specific differences among four large-carnivore species

**Table S1.** Statistics for the radio-telemetry data and locations of predator-killed sheep

This material is available as part of the online article from:

http://www.blackwell-synergy.com/doi/full/10.1111/j.1365-2664.2008.01527.x

(This link will take you to the article abstract).
