# Supplementary material for: Habitat differentiation within the large-carnivore community of Norway's multiple-use landscapes
Source: J Appl Ecol. 2008 Oct;45(5):1382–91. doi: 10.1111/j.1365-2664.2008.01527.x (PMC2658717; doi:10.1111/j.1365-2664.2008.01527.x)
Supplement: Supplementary file 1 [file jpe0045-1382-SD1.doc]

**Appendix S1**: Intra- versus inter-specific differences among four large carnivore species.

We calculated resource selection functions for each individual separately, and used the estimated coefficients to assess inter-specific (species factor), inter-sexual (sex factor) and intra-specific sexual variation (interaction term between species and sex) in habitat selection using multivariate analysis of variance. We found significant inter-specific variation in habitat selection (Pillai’s Trace = 0.940, *F_24,93_* = 1.767, *P* = 0.028), but no inter-sexual variation across species (Pillai’s Trace = 0.188, *F_8,29_* = 0.837, *P* = 0.578) nor intra-specific sexual differentiation (Pillai’s Trace = 0.519, *F_24,93_* = 0.811, *P* = 0.715) in habitat selection was found. Separate models for each species conformed this result, as for none of the species variation in the coefficients could be explained by sex (brown bear: Pillai’s Trace = 0.579, *F_8,11_* = 1.889, *P* = 0.162; lynx: Pillai’s Trace = 0.487, *F_8,7_* = 0.830, *P* = 0.604; wolf: Pillai’s Trace = 0.844, *F_2,1_* = 2.705, *P* = 0.395; wolverine: Pillai’s Trace = 0.269, *F_2,1_* = 0.184, *P* = 0.855).
