# Supplementary material for: Habitat differentiation within the large-carnivore community of Norway's multiple-use landscapes
Source: J Appl Ecol. 2008 Oct;45(5):1382–91. doi: 10.1111/j.1365-2664.2008.01527.x (PMC2658717; doi:10.1111/j.1365-2664.2008.01527.x)
Supplement: Supplementary file 2 [file jpe0045-1382-SD2.doc]

**Table S1.** Statistics for the radio-telemetry data of four carnivore species (habitat) and locations of predator-killed sheep (kill sites) in south-eastern Norway. The rows give the mean and standard deviation for the covariates used in the resource selection functions given in Fig. 2 of the main manuscript.

|  | **Brown bear** | **Wolf** | **Lynx** | **Wolverine** |
| --- | --- | --- | --- | --- |
| *Habitat* |  |  |  |  |
| Elevation (m) | 596 ± 168 | 559 ± 195 | 447 ± 167 | 855 ± 223 |
| Ruggedness | 31 ± 28 | 34 ± 32 | 33 ± 29 | 35 ± 24 |
| Tree cover (%) | 41 ± 16 | 41 ± 18 | 50 ± 18 | 21 ± 17 |
| Distance to forest edge (m) | 85 ± 284 | 133 ± 362 | 104 ± 312 | 633 ± 1,099 |
| Distance to public road (m) | 2,615 ± 2,232 | 2,654 ± 2,393 | 1,648 ± 1,931 | 3,788 ± 1,848 |
| Distance to private road (m) | 394 ± 660 | 357 ± 724 | 113 ± 343 | 1,445 ± 1,345 |
| Distance to building (m) | 753 ± 715 | 636 ± 617 | 365 ± 510 | 1,482 ± 1,023 |
| *Kill sites* |  |  |  |  |
| Elevation (m) | 715 ± 180 | 568 ± 256 | 589 ± 222 | 1,046 ± 174 |
| Ruggedness | 27 ± 21 | 23 ± 17 | 35 ± 29 | 38 ± 24 |
| Tree cover (%) | 34 ± 18 | 42 ± 21 | 40 ± 19 | 10 ± 12 |
| Distance to forest edge (m) | 0 ± 302 | 14 ± 487 | -8 ± 228 | 977 ± 1,330 |
| Distance to public road (m) | 3,552 ± 3,112 | 3,045 ± 4,211 | 1,846 ± 2,852 | 8,033 ± 4,685 |
| Distance to private road (m) | 501 ± 946 | 92 ± 447 | 155 ± 497 | 1,731 ± 1,490 |
| Distance to building (m) | 676 ± 737 | 295 ± 615 | 280 ± 558 | 1,383 ± 1,089 |
